# Rare case of autonomic instability of the lower limb presenting as painless Complex Regional Pain Syndrome type I following hip surgery: two case reports

**DOI:** 10.1186/1752-1947-3-7271

**Published:** 2009-05-29

**Authors:** AJ Shyam Kumar, SKS Wong, JG Andrew

**Affiliations:** 1 All Wales Higher Specialist Training Scheme, Rhos Gwyn, Abergele Road, Colwyn Bay LL29 9AE, UK; 2 Department of Trauma & Orthopaedics, Ysbyty Gwynedd, Penrhosgarnedd, Bangor LL57 2PW, UK

## Abstract

**Introduction:**

According to the International Association for the Study of Pain criteria of 1994, pain is a diagnostic requirement for Complex Regional Pain Syndrome type I. However, other authors have suggested that patients can rarely present with the sensory and vascular symptoms of Complex Regional Pain Syndrome without pain. This entity has not been reported following hip surgery in the English medical literature.

**Case presentation:**

We present two cases of Complex Regional Pain Syndrome-like symptoms following hip surgery and with the total absence of pain. The first case was a 29-year-old Caucasian woman who had a reattachment of the greater trochanter following non-union of an intertrochanteric osteotomy of the hip. Five weeks later, the patient presented with features of Complex Regional Pain Syndrome but with the absence of pain. The second patient was a 20-year-old Caucasian woman who had undergone an open debridement and repair of a torn acetabular labrum. Ten days later, the patient presented with features suggestive of Complex Regional Pain Syndrome which was again painless. Both patients were non-weight bearing at presentation and the symptoms resolved following recommencement of weight bearing.

**Conclusions:**

The authors believe these symptoms are manifestations of vascular changes to the lower limb as a result of non-weight bearing status. Painless Complex Regional Pain Syndrome-like symptoms may occur in patients who are kept non-weight bearing following hip surgery. However, vascular insufficiency and deep venous thrombosis must be excluded before this diagnosis is made. If the clinical situation permits, early weight bearing may relieve symptoms. Orthopaedic and vascular surgeons should be aware of this entity when a postoperative patient presents to them with the above clinical picture. This is also relevant to general practitioners who are likely to see the patients in the postoperative period.

## Introduction

Pain out of proportion to the injury is an essential criterion for the diagnosis of Complex Regional Pain Syndrome (CRPS) type I [1]-[3]. We present two cases of CRPS like symptoms following hip surgery but with the complete absence of pain.

## Case presentation

### Patient 1

**Figure 1:**
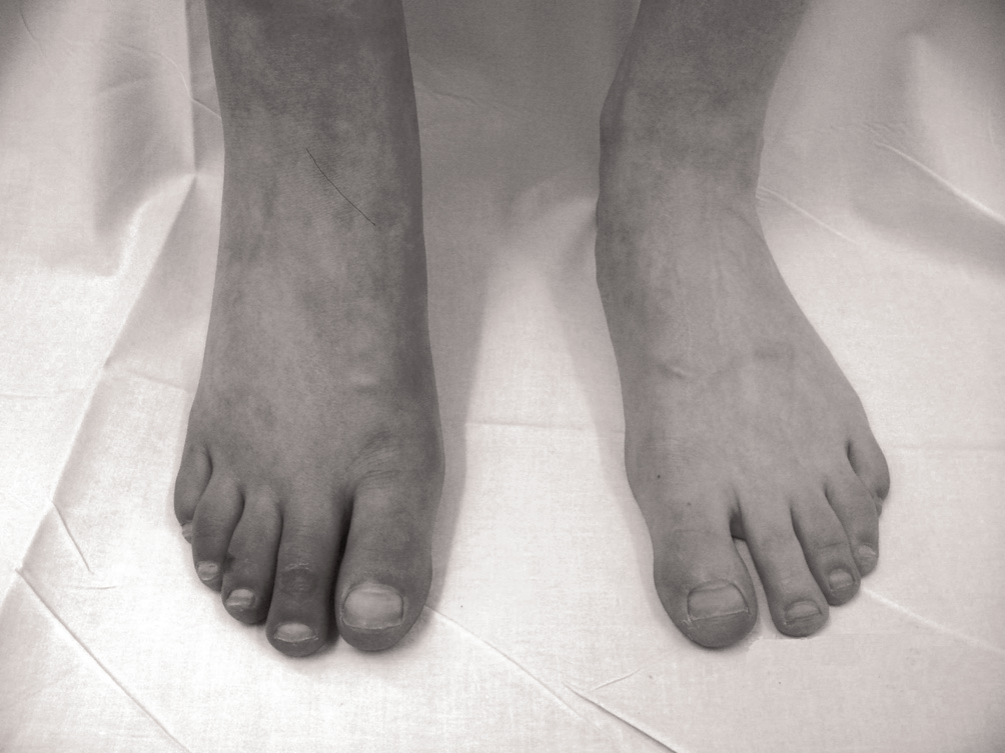
**Right foot shows bluish discolouration compared to the left when it is in the dependent position**. Note the generalized swelling of the right foot with loss of wrinkles compared to the left.

A 29-year-old Caucasian woman had a varus intertrochanteric osteotomy with trochanteric advancement for an old malunited femoral neck fracture. The femoral neck fracture was sustained in a childhood injury and was treated conservatively. The patient underwent reattachment of the greater trochanter for a failed trochanteric fixation approximately 4 weeks after her initial operation. She was discharged 2 days after the second procedure. Approximately 5 weeks later, the general practitioner referred her to the vascular surgeons with painless discolouration of the right leg. Clinical examination revealed a discoloured right foot associated with mild swelling and several small blisters on her toes. The most prominent feature was that the right foot exhibited bluish discolouration which evidently subsided to near-normal colour on elevation (Figure 1). The femoral, popliteal, dorsalis pedis and posterior tibialis pulses were present and normal.

Handheld Doppler examination demonstrated a relative decrease in the audible quality of the biphasic pulse when compared to the normal side. Duplex scan of the right lower limb excluded the possibility of any superficial or deep venous thrombosis. The patient was commenced on 50% weight bearing status and was discharged home. A recent follow-up demonstrated significant improvement in symptoms since commencement of weight bearing status.

### Patient 2

A 20-year-old Caucasian woman who presented with symptoms of right hip impingement underwent open debridement and repair of a torn acetabular labrum. The patient was discharged on the 5th postoperative day following an uneventful recovery period. She was to remain non-weight bearing for 6 weeks. Ten days following discharge, she presented to the clinic complaining of her right foot going blue and cold and with the complete absence of pain. The discolouration occurred when the limb was in a dependent position. Clinically, all peripheral pulses were present and normal. The patient was reviewed by a vascular surgeon who excluded the possibility of arterial insufficiency and deep venous thrombosis.

The patient was discharged with the advice to commence on weight bearing of the operated hip. In the follow-up clinic 5 months later, she was found to have complete resolution of her vascular symptoms.

## Discussion

According to the International Association for the Study of Pain (IASP) criteria of 1994, pain, evidence of change in blood flow or abnormal sudomotor activity and the absence of conditions that would otherwise account for symptoms are essential diagnostic entities of Complex Regional Pain Syndrome type I. Eisenberg and Melamed [4] reported a series of five patients with various foot pathologies who had presented with all of the criteria of Complex Regional Pain Syndrome except pain. The authors are not aware of any English medical literature with reports of painless Complex Regional Pain Syndrome following hip surgery.

Veldman *et al.* reported a series of 829 patients with reflex sympathetic dystrophy (RSD) and among them, 7% of the patients did not have pain as a symptom [5]. Although is not clear from the article if any of the Complex Regional Pain Syndrome-like symptoms developed following hip surgery, this does substantiate the possibility of Complex Regional Pain Syndrome-like symptoms in the total absence of pain.

The authors of this article postulate that in the hip, CRPS-like symptoms develop following a period of non-weight bearing. In both of our patients, the symptoms occurred during non-weight bearing and subsided after weight bearing was commenced. Unilateral lower limb suspension experiments in normal patients have shown an increase in flow mediated dilatation of arteries of the lower limb along with a decrease in venous capacitance [6]. This mechanism may explain the vascular changes which were more intense with the foot in the dependent positions. In such a clinical setting, vascular insufficiency and deep venous thrombosis should be excluded. Unless surgically contraindicated, weight bearing should be commenced at the earliest time possible.

## Conclusion

Painless Complex Regional Pain Syndrome-like symptoms may occur in patients who are kept non-weight bearing following hip surgery. However, vascular insufficiency and deep venous thrombosis must be excluded before this diagnosis is made. If the clinical situation permits, early weight bearing may relieve symptoms.

## Consent

Written informed consent was obtained from the patients for publication of this case report and any accompanying images. A copy of the written consent is available for review by the Editor-in-Chief of this journal.

## Competing interests

The authors declare that they have no competing interests.

## Authors' contributions

JGA postulated the concept and designed the study, interpreted the data and revised the final manuscript. AJSK identified and re-examined the cases, acquired the data and analysed it, reviewed the literature and wrote the article. SKSW also re-examined the cases for the study, assisted in data acquisition, analysed the data and assisted in writing up the article.
